# Targeting SARS-CoV-2 receptors as a means for reducing infectivity and improving antiviral and immune response: an algorithm-based method for overcoming resistance to antiviral agents

**DOI:** 10.1080/22221751.2020.1776161

**Published:** 2020-06-18

**Authors:** Ram Gelman, Areej Bayatra, Asa Kessler, Asaf Schwartz, Yaron Ilan

**Affiliations:** Department of Medicine, Hebrew University-Hadassah Medical Center, Jerusalem, Israel

**Keywords:** Coronavirus, SARS-COV-2, treatment, receptors, ACE, viral resistance, DPP4

## Abstract

The ongoing severe acute respiratory syndrome pandemic caused by the novel coronavirus 2 (SARS-CoV-2) is associated with high morbidity and mortality rates, and it has created a pressing global need for effective antiviral therapies against it. COVID-19 disease pathogenesis is characterized by an initial virus-mediated phase, followed by inappropriate hyperactivation of the immune system leading to organ damage. Targeting of the SARS-CoV-2 viral receptors is being explored as a therapeutic option for these patients. In this paper, we summarize several potential receptors associated with the infectivity of SARS-CoV-2 and discuss their association with the immune-mediated inflammatory response. The potential for the development of resistance towards antiviral drugs is also presented. An algorithm-based platform to improve the efficacy of and overcome resistance to viral receptor blockers through the introduction of personalized variability is described. This method is designed to ensure sustained antiviral effectiveness when using SARS-CoV-2 receptor blockers.

## 1. Introduction

The ongoing severe acute respiratory syndrome pandemic caused by the coronavirus 2 (SARS-CoV-2), which causes the coronavirus disease (COVID-19), is associated with high morbidity and mortality rates worldwide [1]. Two previous coronaviruses have already previously drawn global attention, by causing potentially lethal epidemic outbreaks: the severe acute respiratory syndrome coronavirus (SARS-CoV), and the Middle East respiratory syndrome coronavirus (MERS-CoV) [2]. Currently, there are no specific antiviral drugs or vaccines for treatment of COVID-19 [3]. Moreover, the potential for resistance to antiviral agents, as is common in numerous viruses, may become a major obstacle for the development of effective therapies against SARS-CoV-2 [4].

In the present paper, we outline the potential targeting of receptors for SARS-CoV-2 therapy. The potential effect of blocking these receptors in the modulation of downstream immune responses, both in initial and late phases of the disease, is discussed. An algorithm-based platform to improve the efficacy of and overcome resistance to viral receptor blockers by introducing personalized variability is presented.

### 1.1. SARS-CoV-2 pathogenesis, infectivity, and target organ damage

SARS-CoV-2 infects lung cells after being transmitted via person-to-person transmission, even while the carrier is asymptomatic [5]. The pathogenesis of COVID-19 depends on the interactions between the virus and the immune system [6].

Coronavirus tropism is predominantly determined by interactions between the viral spike (S) proteins and host receptors [7]. Cell entry of coronaviruses requires the binding of the viral S protein to cellular receptors and depends on S protein priming by host cell proteases [8]. The spike protein contributes to host receptor binding, cell tropism, and pathogenesis, and it acts by binding to host receptors on target cells and inducing endocytosis of virions. This is followed by the fusion of host and viral membranes, allowing for the penetration of the viral genome into the host cytoplasm. The S protein is also a target of the host immune system, which adds selective pressures to this biochemical machinery [9]. Coronavirus spikes can recognize a broad range of cell-surface molecules in addition to target receptors, thereby augmenting coronavirus cellular attachment and entry [7].

COVID-19 disease progression follows a two-step process. In the viral infection phase, cellular infection takes place in various organs via specific receptors [10]. Initially, symptoms that present are constitutional such as fever, myalgia, and respiratory symptoms including throat pain, cough, and shortness of breath [11]. The innate immune response is mediated by interferon α (IFNα) secretion and characterized by elevated levels of interleukin 6 (IL-6), CRP, and neutrophils, with an accompanying decrease in lymphocyte count. The virus can hamper IFN production and downstream signaling, and a dysregulated type I interferon response is part of the pathogenesis of severe infections [12].

The initial viral-infection phase is followed by an inappropriate hyperactivation of the immune response, involving multiple cytokines and immune cells, which induce immune-mediated end-organ damage [12]. In some patients, the disease progresses to a severe form which most commonly manifests as acute respiratory distress syndrome (ARDS) followed by respiratory failure, acute myocardial injury, cardiac dysfunction, shock, and multiple organ failure [5]. The severe form of the disease is associated with increased cytokine levels (IL-6, IL-10, and TNFα), lymphopenia (in CD4+ and CD8+ T cells), and decreased IFNγ expression in CD4+ T cells [13]. Viral binding to the toll like receptor (TLR) promotes pro-IL-1β cleavage by caspase-1, followed by inflammasome activation and an IL-1β surge that induces lung inflammation, fever, and fibrosis [14].

The adaptive immune response towards SARS-CoV-2 is a Th1 type response, mediated by cytotoxic T cells (responsible for killing virus-infected cells), and a humoral response comprising antibody production to neutralize the virus and ultimately protect from the disease [15]. A Th1 response is associated with stronger levels of T cell activity and neutralizing antibodies, leading to recovery, while a Th2 response may be associated with fatal disease [16].

Taken together, the data support the presence of virus-immune system interactions, which underlie the pathogenesis of COVID-19.

### 1.2. ACE2 is a SARS-CoV-2 receptor that may affect the antiviral immune response

The renin-angiotensin system (RAS) is essential for the regulation of organ functions including those relating to the cardiovascular system, blood pressure, fluid and electrolyte balance, the kidneys, and the lungs. RAS is also known to exert tissue-specific local effects associated with hypertension, myocardial injury, heart failure, diabetes, and inflammatory lung diseases [17]. RAS and the angiotensin-converting enzyme (ACE) are subjects of interest in determining the pathophysiology of lung inflammation in numerous disease processes [18]. Renin secretion from the juxtaglomerular apparatus of the kidneys in response to a variety of stimuli acts on the circulating precursor angiotensinogen to generate angiotensin I. The conversion of angiotensin I, which lacks vasoconstriction properties, to angiotensin II, a potent vasopressor, is due to the action of ACE [19]. The modulation of RAS via ACE inhibition has been a primary strategy in the treatment of hypertension and heart failure [19].

ACE2, a human ACE homologue, is found primarily in the heart, kidneys, and testicular tissues in humans [20]. ACE2 has a deceptive signaling peptide, a metalloprotease active site, and a transmembrane domain, which share genomic structures with ACE, suggesting that the two genes share a common ancestor. ACE2 is a negative RAS regulator, and ACE/ACE2 imbalance is an important parameter in several disease processes including lung injuries associated with ARDS [21,22].

ACE2 is a metallopeptidase that serves as a cellular entry point for SARS-CoV and is more abundantly expressed on the apical surface than the basolateral surface of polarized airway epithelia, as well as by oral mucosal epithelial cells [23,24]. ACE2 expression positively correlates with the differentiation state of epithelia. Undifferentiated cells expressing low ACE2 levels are poorly infected by SARS-CoV, while well-differentiated cells expressing high levels are readily infected [25]. A high affinity between ACE2 and the viral S1 protein domain of the SARS-CoV S protein has been described. Viral entry into and replication in host cells depends upon interactions between the viral S protein, and the ACE2 receptor on the host cell membrane. This is followed by S protein priming of the transmembrane protease serine 2 (TMPRSS2) [8,26].

The ability of anti-ACE2 antibodies, but not anti-ACE antibodies, to inhibit viral replication in susceptible cells has been shown in a dose-dependent manner, which supports a substantial contribution of ACE2 to the efficiency of SARS-CoV replication [23]. Attenuation of RAS activity protects the lungs and is dependent on ACE2 expression. By deregulation of this lung-protective pathway, the virus is thought to exhibit higher lethality [27]. ACE2 is also expressed by gastrointestinal epithelial cells, suggesting that SARS-CoV-2 can actively infect and replicate in the gastrointestinal tract [28].

There are multiple similarities between SARS-CoV-2 and SARS-CoV. The three dimensional structures of the spike protein receptor-binding domain of both viruses are almost identical, with a high degree of homology and 76% amino-acid sequence identity similarity [29]. Like other coronaviruses, SARS-CoV-2 uses the S protein as the main interacting protein with host cell receptors, including the SARS-CoV receptor ACE2 for entry, and the serine protease TMPRSS2 for S protein priming [16]. A TMPRSS2 inhibitor inhibited the viral entry and sera from convalescent SARS patients cross-neutralized SARS-CoV-2 S-driven entry [8]. SARS-CoV, and presumably SARS-CoV-2, leads to downregulation of the ACE2 receptor, but not the ACE receptor, via binding of the S protein with ACE2. This leads to viral entry and replication, and potentially to severe lung injury [27]. The receptor-binding domain (RBD) of the SARS-CoV-2 S protein binds to ACE2 receptors, and it manifests a higher ACE2 binding affinity than that of SARS-CoV, and it could block the binding and attachment of SARS-CoV-2 RBD to ACE2-expressing cells inhibiting their infection to the host cells [30].

Patients with cardiovascular and respiratory diseases such as hypertension, heart failure, and obstructive or inflammatory lung disease are more susceptible to SARS-CoV-2 infection [31]. This may be related to differences in RAS activation and ACE2 expression, in addition to upregulation of the receptor in the lungs in response to medical treatment that contributes to RAS inhibition [32,33].

Glutamyl aminopeptidase (ENPEP) is a member of the M1 family of endopeptidases, which are membrane type II zinc-containing endopeptidases. It is involved in the catabolic pathway of RAS-formed angiotensin III, and it has been proposed as another potential receptor for human coronaviruses [34].

Potential therapeutic approaches have been proposed to target various steps in the viral infectious process, including a SARS-CoV-2 S-protein-based vaccine, a TMPRSS2 inhibitor aiming to block the priming of the viral S protein, an anti-ACE2 antibody to block the surface receptor, and a soluble ACE2 analogue which competitively binds with SARS-CoV-2 to slow viral entry into cells and decrease viral spread [27].

ACE2 also plays a role in immune function and may be linked to immune responses associated with COVID-19 pathogenesis. RAS is associated with inflammation and fibrosis, and the RAS axis is composed of ACE2 and angiotensin-(1-7), while the Mas receptor exerts opposite effects in terms of inflammatory responses and tissue fibrosis. Viral respiratory infections such as influenza strains, the respiratory syncytial virus, and SARS-CoV-2 can mediate lung inflammation and injury via ACE2 dysregulation [35,36].

Downregulation of ACE2 activity in mice treated with an ACE2 inhibitor prior to instilling lipopolysaccharide (LPS) was shown. It-facilitated neutrophil infiltration parallel to ACE2 reduction, via reduced ACE2 inhibition of des-Arg9 bradykinin (DABK)/bradykinin receptor B1 (BKB1R) axis signaling. This led to pro-inflammatory chemokine expression, increased neutrophil infiltration, and exaggerated levels of lung inflammation and injury [37]. These effects affect additional inflammatory processes in the lungs, including pulmonary fibrosis, chronic obstructive pulmonary disease (COPD), asthma, and acute lung injury due to potentiation of pro-fibrotic and pro-inflammatory cytokines and stimulation of angiotensin II receptors. These processes are associated with the expression of pro-inflammatory mediators, such as interleukin(IL)-8/cytokine-induced neutrophil chemoattractant-3 and IL-6 [38].

The anti-inflammatory and anti-fibrogenic effects of the ACE2/Ang-(1-7)/Mas axis are linked to reduced cytokine release and the inhibition of fibrosis-associated signaling pathways in models of atherosclerosis, cerebral ischemia, obesity, chronic kidney disease, liver disease, and asthma [39]. Loss of ACE2 is associated with increased neutrophil, macrophage, and T-cell infiltration in a model of acute kidney injury. MRNA levels relating to pro-inflammatory cytokines, IL-1β, IL-6, TNFα, the macrophage inflammatory protein 2, and the monocyte chemoattractant protein-1, were increased in ACE2 knockout (KO) mice. Decreased ACE2 expression is associated with increased apoptosis and oxidative stress [40]. Amelioration of sepsis-induced-lung injury is mediated via regulation of the ACE2/Ang-(1-7)/Mas axis and inhibition of the MAPK/NF-kappaB inflammatory signaling pathways [41]. Treatment with an angiotensin I receptor blocker prevented angiotensin II-mediated aortic profilin-1 expression, and inflammation [42].

Clinical data suggested that application of ACE inhibitors and ARB contribute to the clinical outcomes of COVID-19 patients with hypertension. Patients receiving ACEI or ARB therapy had a lower rate of severe diseases, a trend toward a lower level of IL-6 in peripheral blood, and decreased peak viral load [43]. Among hospitalized COVID-19 patients with hypertension, the use of these drugs was associated with lower risk of all-cause mortality [44].

These data suggest that, under some conditions, worsening of inflammation occurs using ACE2 blockers, which may be beneficial in early stages of SARS-CoV-2 infections. The effects of these blockers during late disease phases, in which the target organ damage is mediated by inflammatory pathways, remain to be shown.

### 1.3. Dipeptidyl peptidase 4 is a coronavirus receptor, which may also be associated with the antiviral immune response

Dipeptidyl peptidase 4 (DPP4), also known as CD26, is a 110 kDa transmembrane glycoprotein expressed on the surface of a wide variety of epithelial cells and some lymphocytes. It acts as a peptidase, cleaving N-terminal dipeptides and degrading numerous substances including hormones, neuropeptides, chemokines, and cytokines. The most notable of these are the incretin hormones, which participate in glucose metabolism [45].

DPP4 is uncommon in surface epithelial cells from the nasal cavities to the conducting airways, with a somewhat increased incidence in the distal airways. It has been detected in mononuclear leukocytes and serous cells of submucosal glands [46]. In the lungs, it is present on the surfaces of non-ciliated bronchial epithelial cells, on type I and II cells, alveolar macrophages, and on vascular endothelial cells, lymphatics, and pleural mesothelia. Patients with chronic lung disease manifest increased DPP4 immunostaining in alveolar epithelia, type I and II alveolar cells, and alveolar macrophages [46].

DPP4 is a known receptor for MERS-CoV, and antibodies against it inhibit hCoV-EMC infection of primary human bronchial epithelial cells and Huh-7 cells [47].

A study that examined the SARS-CoV-2 S protein suggested that its S1 domain, which presumably serves as the viral binding site, interacts with DPP4 [48,49]. SARS-CoV-2 is thought to have a zoonotic origin prior to person-to-person transmission, and it has a high genetic similarity to SARS-CoV and MERS-CoV, which both originated in bats [50,51]. The DPP4 protein ‘s amino acid sequence is highly conserved across different species, including bats. In vitro expression of human and bat DPP4 in previously non-susceptible cells without previous DPP4 expression subsequently enabled infection of those cells by MERS-CoV. Serious adverse outcomes in patients with SARS-CoV-2 have been described more frequently in patients with COPD comorbidity [52]. Smokers, and subjects with COPD, express higher rates of DPP4 [53].

DPP4 is also expressed on immune cells including T and B lymphocytes, activated natural killer (NK) cells, and myeloid cells. It plays a role in T-cell mediated immune responses, and contributes to T cell development, maturation, differentiation and activation [54]. DPP4 is involved in T-cell co-stimulatory activation via its association with adenosine deaminase (ADA), caveolin-1, the caspase recruitment domain-containing membrane-associated guanylate kinase protein-1 (CARMA-1), CD45, the mannose-6-phosphate/insulin growth factor-II receptor (M6P/IGFII-R), and the C-X-C motif receptor 4 (CXC-R4). DPP4 mediates co-stimulation in human CD8+ lymphocytes, contributing to acquired immune responses, and its role in T-cell immunity is attributed to its peptidase activity and ability to serve as a receptor for numerous molecules, including memory antigens [55].

DPP4 inhibitors modulate TCR signaling, inhibit the proliferation of CD4+ and CD8+ lymphocytes, and suppress antigen-stimulated CD4+ T-cell clone secretion of IFN-γ, TNF-α, and IL-4 in a dose-dependent manner [56]. DPP4 is also a positive regulator of B cell activation, and its inhibitors suppress that activation via suppression of DNA synthesis in mitogenic active B cells [57].

The interaction of the MERS-CoV S protein with DPP4 initiates signals that suppress macrophage activation [58]. Infection of macrophages with particles pseudotyped with the MERS-CoV S protein also suppresses macrophage responses, by reducing their capacity to produce TNFα and IL-6 in naive and LPS-activated THP-1 macrophages, and by augmenting the LPS-induced production of the immunosuppressive cytokine IL-10. The MERS-CoV S protein induces the expression of a negative regulator of TLR signaling, IRAK-M, and of a transcriptional repressor, PPARγ [58].

Sitagliptin is a DPP4 inhibitor used for treatment of diabetes mellitus. It can inhibit proliferation of phytohemagglutinin-stimulated peripheral blood mononuclear cells (PBMC) from healthy volunteers, decreased CD26 expression, and reduced the proportions of Th1, Th2, and Th17 lymphocytes [59]. Clinical treatment with sitagliptin reduced the number of circulating CD4+ T cells, Th17 lymphocytes, and regulatory T cells, and increased the number of circulating CD34 CXCR4 cells by approximately a factor of two in patients with type 2 diabetes [60]. Inhibition of DPP4 by siRNA or sitagliptin diminished the effects of the MERS-CoV S protein on IRAK-M, PPARγ and IL-10, supporting its immunosuppressive effects [58].

**Figure 1.**
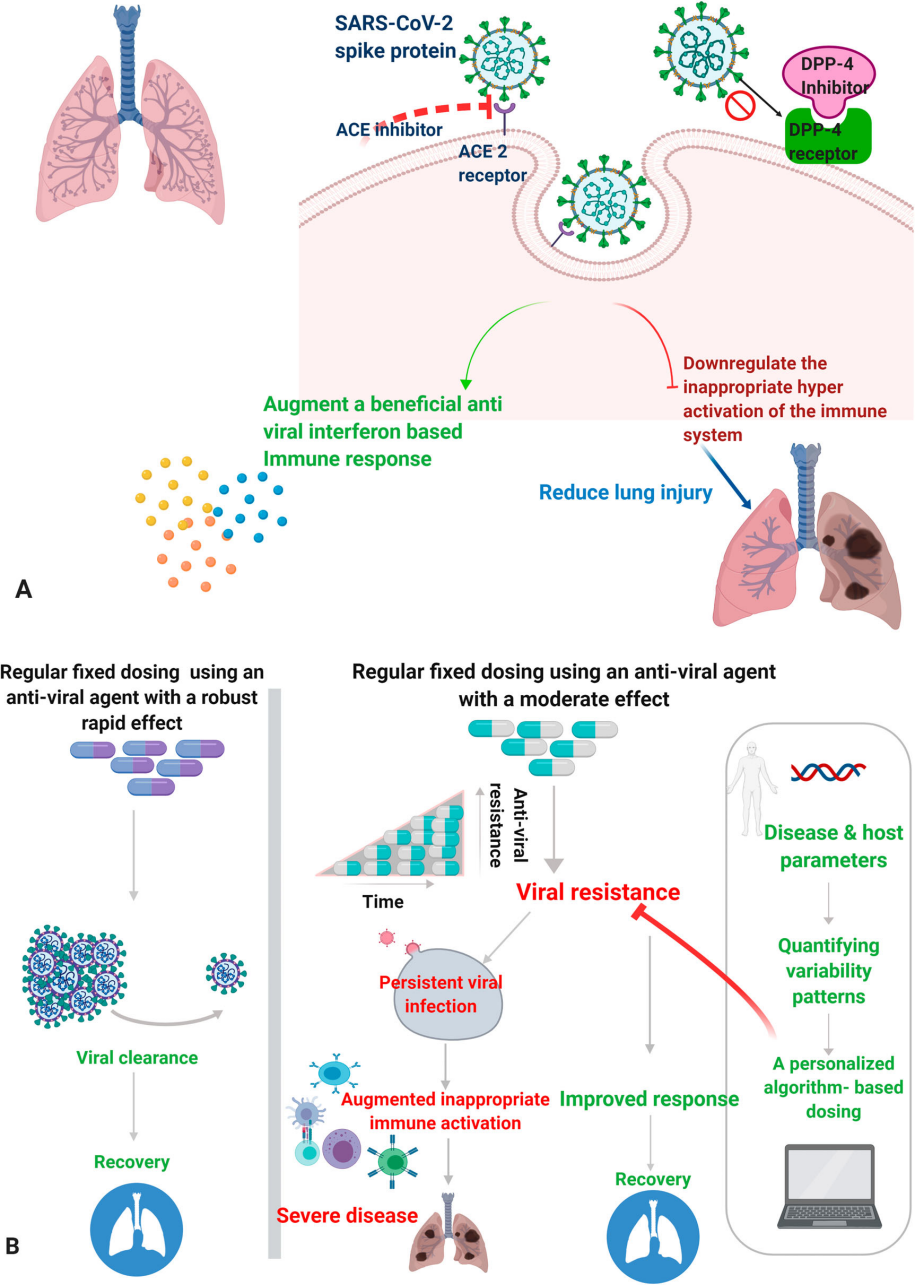
**A.** Potential targets for treatment of SARS-CoV-2 infection at the receptor level affecting downstream immune responses to the virus. **B.** The introduction of personalized variability to counter resistance to antiviral treatments. Regular, fixed dosing using an antiviral agent with robust and rapid effects is expected to lead to viral clearance, while fixed dosing regimens of moderately effective drugs may lead to drug resistance, viral persistence, an augmented hyperactivation of the immune system, and severe disease. The introduction of an algorithm-based dosing method based on quantified variability patterns derived from disease and host parameters improves the response to antiviral drugs.

The data suggest that blocking DPP4 receptors may have potential benefits in modulation of the COVID-19 immune response, both in early and late phases of the disease. Sitagliptin has been proposed as a potential therapy for patients infected with SARS-CoV-2. Figure 1A shows a schematic representation of the effects of blocking ACE2 and DPP4 receptors on viral infectivity, and on immunomodulation of the inappropriate immune hyperactivation that induces the organ damage during the disease.

### 1.4. The SARS-CoV-2 spike-host cell receptor GRP78 binding site

The glucose-regulated protein 78 (GRP78) is an endoplasmic reticulum (ER) chaperone that plays a role in viral entry [61,62]. It is involved in protein folding and assembly, translocation of newly synthesized polypeptides, degradation of misfolded proteins, and the maintenance of ER homeostasis [63]. GRP78 is a regulator of ER stress due to its role in the unfolded protein response pathway, and it has also been detected in the mitochondria, nucleus, cytosol, and plasma membrane. It is associated with cell surface regulation of signaling and cellular homeostasis [64]. In the ER lumen, it binds to and inactivates three enzymes responsible for cell death or differentiation, including activating transcription factor 6 (ATF6), protein kinase RNA-like endoplasmic reticulum kinase (PERK), and the inositol-requiring enzyme 1 (IRE1) [86]. Above a certain threshold of accumulated unfolded proteins, GRP78 releases and activates ATF6, PERK, and IRE1, leading to the inhibition of protein synthesis and enhancement of protein refolding [64]. Overexpression of GRP78 is initiated by cellular exposure to stress, which increases the likelihood that it will escape ER retention and translocate to the cell membrane. Once there, its substrate-binding domain (SBD) can be recognized by the virus, which allows viral entry into the cell. GRP78 inhibits hepatitis B virus replication by reducing viral production and protein secretion [65].

GRP78 is abundantly expressed by epithelial cells of the bronchus, bronchiole, and alveolus. Double immunostaining of DPP4 and GRP78 demonstrated the co-localization of DPP4 and GRP78 in the epithelial cells lining the human airways. GRP78 augments viral entry to susceptible cells in the presence of the host cell receptor DPP4 [8]. The co-localization of DPP4 and GRP78 to the epithelial cell apical sides further supports the notion that GRP78 facilitates MERS-CoV entry or attachment. GRP78 assists in the attraction of virus particles to the cell surface, increasing the likelihood of receptor-mediated MERS-CoV and bat coronavirus HKU9 (bCoVHKU9) viral entry. MERS-CoV infection results in an up-regulation of GRP78 on the cell surface, which increases viral attachment and enhances viral entry into infected cells [7].

Infections by certain coronaviruses, including the infectious bronchitis virus and SARS-CoV, induce ER stress leading to GRP78 expression on the cell surface [66,67]. The SARS-CoV S protein induces transcriptional activation of GRP78, and a substantial amount of S protein accumulates in the ER. The expression of the S protein exerts different effects on the three major signaling pathways of the unfolded protein response (UPR), inducing GRP78 through PKR-like ER kinase [68].

A recent study proposed that the SARS-CoV-2 S protein binds to the GRP78 cell-surface receptor [69]. The SARS-CoV-2 S protein has been modelled using its counterpart, the SARS-CoV spike protein. Pep42 is a cyclic peptide that binds to GRP78 at the surface of cancer cells. Sequence and structural alignments have shown that four regions of the S protein have sequence and physicochemical similarities to Pep42. Protein–protein docking studies have shown that the four S protein regions fit tightly in the GRP78 substrate binding domain β (SBD β). The docking pose showed an association of the SBD β of GRP78 and the receptor-binding domain of the virus S protein in recognition of the host cell receptor. Region IV predicted a high binding affinity, and it has been proposed as a potential therapeutic target [69].

### 1.5. Resistance to antiviral agents is a major obstacle to sustainable therapeutic effectiveness

Similarly to SARS-CoV and MERS-CoV, there is no clinically proven specific antiviral agent available to treat SARS-CoV-2 infections. Several potential therapies, including the repurposing of existing antiviral agents, have been proposed [70]. The broad-spectrum antiviral remdesivir and chloroquine have been effective for viral control *in vitro*. Remdesivir is an adenosine analogue which targets the RNA-dependent RNA polymerase and blocks viral RNA synthesis, and it has shown antiviral activity against a wide array of RNA viruses including SARS/MERS-CoV. Other nucleoside analogues, such as favipiravir, ribavirin and galidesivir have also been suggested as potential treatments [70,71]. Studies supporting some role for broad-spectrum antiviral remdesivir and chloroquine in COVID-19 were published [72–81].

Prolonged exposure to antiviral drugs and ongoing viral replication are key factors in the development of drug resistance, which may manifest as persistent or increasing viremia or severe disease resulting from progressive viral infection. In cases where the treatment course is effective and viral fitness is impaired sufficiently, no viral genomes will be successfully replicated. However, in conditions where the treatment is not as effective and some genomes replicate, selective pressures may result in rapid adaptation leading to viral resistance [82].

Both host and viral factors are linked to the development of resistance, which involves mechanisms of viral replication, genomic inference, or selective pressures that result in viral adaptation associated with high rates of viral mutations. Antiviral resistance is linked to high mutation and recombination rates, demographic histories of transmission, compartmentalization, and selective forces incurred during viral adaptation to drugs [82].

Both receptor-level and post-receptor-level resistance may occur. Antiviral inhibition of the viral replication cycle can occur at the level of virion inhibition, adsorption, viral penetration, viral uncoating, genome replication, or the release of mature virions [83]. The process of entry into a host cell is important for the life cycle of most viruses, and broad-spectrum antiviral approaches which target host cell proteins and pathways are being used. Following the attachment to the cell surface or receptors, many viruses induce changes to environmental conditions, such as pH, interactions with a cellular receptor, or modulate the activity of proteolytic enzymes which leads to conformational changes in key proteins that mediate cell membrane penetration [84].

Models have been developed to describe the development of influenza resistance using semi-stochastic simulations which determine the emergence of resistant mutants during infection, in the presence or absence of antiviral agents. A mismatch between surface proteins and internal RNA correlated with a reduced likelihood of the appearance of drug-resistant mutants. Late occurrence of mutants was thought to be because the mismatch provided an option to prevent the propagation of the mutation. The immune response may also lower the probability of drug-resistant infection [85].

Resistance against protease or polymerase inhibitors used for the treatment of hepatitis C viral infections have been linked to a lower genetic barrier to resistance, and they can be achieved through a single or a small number of mutations. Combinations such as ledipasvir and sofosbuvir, which have high genetic barriers to resistance, show little cross-resistance between the two drugs. Acyclovir and its related analogues used against herpes viruses are nucleoside inhibitors, against which resistance mutations are known that affect either a thymidine kinase required for prodrug activation by phosphorylation, or the DNA polymerase. HIV reverse transcriptase is highly error prone, leading to a high rate of nucleotide substitutions, increased population diversity, and frequent resistance mutations and drug resistance. Combination therapies reduce the incidence of resistance without known cross-resistance mutations [86].

The overcoming of drug resistance, especially under conditions where the drugs are unable to provide rapid and complete viral clearance, is a major obstacle for improving the morbidity and mortality rates caused by numerous viruses. The use of an integrative antiviral drug repurposing methodology has been proposed to implement a pharmacology-based platform able to shorten the time to develop anti-SARS-CoV-2 drugs [87]. However, these may not only fail to prevent resistance, but may actually promote it due to the relatively moderate antiviral effects of these agents.

### 1.6. The introduction of variability into therapy to overcome resistance at the receptor level

Biological systems are characterized by an inherent variability, which can be viewed as a property of causal processes, contributing to their function. The stochastic behaviour of these systems typifies their dynamic behaviour in response to internal and external triggers. Intrinsic stochasticity has been described for intracellular pathways [88]. Intra- and inter-subject biological variability has been described at the cellular organelle level, as well as for whole organs [88]. Variability also underlies responses to disease-inducing triggers and also to the effect of drugs [89].

Individual tolerance to drugs is a random variable that derives from multiple genetic and environmental factors, which suggests that deterministic equations are not suitable for modelling it. Variability of the treatment response has been attributed to pharmacogenomic- and pharmacodynamic-based drug metabolism parameters, but the heterogeneity in the responses cannot be attributed solely to these factors [90]. Complex intracellular drug-target and drug-receptor interactions have been described. Non-specific interactions slow the incorporation kinetics of DNA-binding drugs, and have been attributed to irregular drug diffusion in cells. In humans, marked intra-patient variability in drug serum levels between days has been described [91]. The unpredictability of drug effectiveness is partly associated with the dynamicity and continuously changing rules by which these systems function. Dosing regimens of drugs, which are based on regular fixed schedules, have been proposed to be incompatible with the physiological variability by which these systems operate, leading to primary or secondary loss of response [92,93].

Chronic anti-TNFs therapy is associated with high rates of drug response loss. Dosage escalations and reductions, as well as drug holidays, are used in real-world settings. Intermittent dosing with drug holidays has shown clinical benefits while helping to minimize drug exposure and potential adverse effects. In a prospective trial of patients with inflammatory bowel disease, irregular dosing reduced the loss of response compared to regular fixed dosing [94].

Antiviral resistance at the receptor or post-receptor levels may be overcome by the implementation of personalized variability patterns in treatment regimens. A patient-tailored approach, which implements personalized variability patterns into treatment algorithms, has been proposed to improve the treatment sustainability. Patterns of variability can be quantified based on host and disease-related variability patterns, immune tests, genetic profiling, chronotherapy, heart rate variability, and additional parameters [92,93]. Algorithm-controlled treatment regimens are now being used in several clinical trials for overcoming drug resistance (NCT03843697; NCT03747705).

A similar platform has been proposed to ensure sustained effects of receptor-targeted therapies for SARS-CoV-2 infections. Figure 1B shows a schematic representation of the effect of the introduction of personalized variability as a counter to antiviral drug resistance. Regular, fixed dosing using an antiviral agent with robust and rapid effects is expected to aid viral clearance and recovery. On the other hand, fixed dosing with a moderately effective antiviral agent may lead to viral resistance and persistent infection. Viral persistence is expected to further augment the immune hyper-activation, leading to worsening of disease severity. The introduction of an algorithm-based variable treatment approach, which quantifies disease and host-related personalized patterns, can overcome drug resistance and improve treatment responses.

## 2. Conclusion

In summary, the high morbidity and mortality rates in patients with COVID-19, and the lack of effective treatments, make them a worldwide challenge. Targeting of potential receptors has been proposed to develop receptor-targeted therapies against the virus. These blockers have the potential both to reduce infectivity and to counter the inappropriate over-activation of the immune system that is responsible for end organ damage. As the likelihood of developing a powerful agent, which can rapidly clear the virus, is low, drug resistance is a major obstacle for drug development. Administration of antivirals using an algorithm-regulated regimen can improve the efficacy of these therapies, preventing the development of resistance and ensuring sustained effects. Future clinical trials will explore the effect of using these personalized algorithms to improve the clinical effects of antiviral agents.

## Abbreviations

Abbreviations: SARS-CoV-2: severe acute respiratory syndrome coronavirus 2; IFNα: S: spike protein; RAS: renin angiotensin system; Interferon α; IL-6: interleukin 6; ARDS: acute respiratory distress syndrome; IL-10: interleukin 10; TNFα: tumor necrosis factor alpha; IFNγ: interferon gamma; TLR: Toll Like Receptor; IL-1β: interleukin-1 beta; ACE: angiotensin-converting enzyme; RBD: receptor-binding domain; TMPRSS2: transmembrane protease serine 2; S protein: spike glycoprotein; LPS: lipopolysaccharide; COPD: chronic obstructive pulmonary disease; IL-8: interleukin 8; NF-kappaB: nuclear factor–kappa B; ARB: angiotensin receptor blocker; DPP4: Dipeptidyl peptidase 4; MERS-CoV: Middle Eastern respiratory syndrome coronavirus; SARS-CoV: Severe Acute Respiratory Syndrome Coronavirus; NK: natural killer; IGFII-R: insulin growth factor-II receptor; CXC-R4: C-X-C motif receptor 4; PBMC: peripheral blood mononuclear cells; GRP78: Glucose-regulated protein 78; ER: endoplasmic reticulum; ATF6: activating transcription factor 6; PERK: protein kinase RNA-like endoplasmic reticulum kinase; IRE1: inositol-requiring enzyme 1; SBD: substrate-binding domain; SBD β: substrate binding domain β; COVID-19: coronavirus disease-19;
